# Social Contact Reinforces Cocaine Self-Administration in Young Adult Male Rats: The Role of Social Reinforcement in Vulnerability to Drug Use

**DOI:** 10.3389/fnbeh.2021.771114

**Published:** 2021-10-29

**Authors:** Mark A. Smith, Hannah S. Cha, Annie K. Griffith, Jessica L. Sharp

**Affiliations:** Department of Psychology and Program in Neuroscience, Davidson College, Davidson, NC, United States

**Keywords:** addiction, preclinical model, social influence, social learning, substance use disorder

## Abstract

Drug-using peers are recognized as a leading factor influencing drug use among adolescents and young adults. One mechanism by which peers influence drug use is by providing social reinforcement for using drugs. Social reinforcement may be provided in multiple ways, including by making social contact contingent on drug use (i.e., an individual must use drugs to gain/maintain access to a peer). The purpose of this study was to develop a preclinical model in which intravenous cocaine self-administration was positively reinforced by access to a social partner. Young adult male rats were trained to self-administer cocaine in operant conditioning chambers with a guillotine door that could be opened to an adjacent compartment housing either a social partner or a non-social stimulus. Once cocaine self-administration was established, the guillotine door was activated, and cocaine intake was reinforced by brief access to either a social (age- and sex-matched peer) or non-social (black-and-white athletic sock) stimulus. Contingent access to a social partner rapidly increased cocaine self-administration. Total cocaine intake was 2- to 3-fold greater in rats assigned to the social versus non-social condition across a 100-fold dose range. Cocaine intake rapidly increased when rats in the original non-social group were later provided with social partners, whereas cocaine intake resisted change and remained elevated when rats in the original social group had their partners removed. These data indicate that contingent access to a social partner increases drug intake and suggest that social reinforcement may represent a vulnerability factor that is particularly resistant to psychosocial interventions.

## Introduction

Epidemiological studies reveal that one of the most reliable predictors of whether an adolescent or young adult will use drugs is whether his or her friends use drugs (Wills and Cleary, 1999; Bahr et al., 2005; Simons-Morton and Chen, 2006; Tompsett et al., 2013; Barnett et al., 2014; Schuler et al., 2019). Consequently, drug use among an individual’s peers represents a major vulnerability factor determining whether an individual will use drugs and develop a substance use disorder. Theoretical approaches to explain the high concordance rate of drug use among peers have focused on the roles of (1) selection, in which an individual self-selects peers based on shared interests (e.g., drug use), and (2) social learning, in which peers establish and maintain drug use amongst one another *via* associative learning mechanisms (see reviews by Kandel, 1986; Andrews and Hops, 2010; Pandina et al., 2010 further discussion of selection and socialization theories). Although these theoretical approaches are not mutually exclusive, only the latter lends itself to behavioral interventions that may reduce drug use among vulnerable populations.

Social learning models of drug use posit that drug use is established and maintained by contingencies operating in an individual’s social environment (Akers, 1977; Strickland and Smith, 2014). For instance, drug use is established by observing and modeling the behavior of a peer using drugs, and drug use is maintained by social reinforcement provided by the peer. This reinforcement could be in the form of verbal encouragement or simply by continued access to the peer. Unfortunately, empirical support for the role of social learning in drug use is limited. Ethical constraints limit the degree to which drug use, particularly illicit drug use, can be modeled and reinforced in humans, and animal models, particularly those that use intravenous drug self-administration, have traditionally been limited by the necessity of testing animals in isolation.

Recently, several studies have described the use of modified operant conditioning chambers that permit one or more animals to intravenously self-administer drugs in proximity to a social partner, which has rapidly advanced our understanding of how social contact can increase or decrease drug intake. For instance, these studies have shown that drug intake is increased in the presence of a partner self-administering drugs (Smith, 2012; Smith et al., 2014; Robinson et al., 2016, 2017), drug intake is decreased in the presence of a partner without access to drugs (Smith, 2012; Smith et al., 2014; Peitz et al., 2013; Robinson et al., 2016, 2017), patterns of drug intake between partners become more similar over time (Lacy et al., 2014), and subjects will maintain voluntary abstinence when given a choice between drugs and access to a social partner (Venniro et al., 2018, 2019, 2021). Importantly, social contact serves as a positive reinforcer in laboratory animals, and contingent access to a social partner can establish responding in experimentally naïve rats (Angermeier, 1960) and maintain rates of responding similar to those of consummatory reinforcers (e.g., food; Evans et al., 1994). The extent to which social reinforcement increases drug intake in these models has not been examined.

The aim of this study was to establish an animal model in which drug intake is positively reinforced by access to a social partner. To this end, male rats were implanted with intravenous catheters and trained to self-administer cocaine in modified operant conditioning chambers in which a solid guillotine door could be opened to an adjacent compartment housing either a social partner or a non-social (i.e., negative control) stimulus behind a metal screen. Once cocaine self-administration was established, the guillotine door was activated, and cocaine intake was reinforced by brief access to either the social or non-social stimulus. We predicted that cocaine intake would be greater in the social than non-social condition, thus supporting a role for social reinforcement in the facilitation of drug use.

## Materials and Methods

### Subjects

Male, Long-Evan rats were obtained on postnatal day 49 and housed individually in polycarbonate cages in a temperature- and humidity-controlled colony room maintained on a 12:12 h light-dark cycle. All rats had access to bedding, enrichment materials (e.g., gnaw sticks, plastic enclosures), and water throughout the study. Food was available *ad libitum*, except during the brief period of lever press training described below. All subjects were maintained in accordance with the requirements of the Davidson College Animal Care and Use Committee and the guidelines described in the *Guide for the Care and Use of Laboratory Animals* (Institute for Laboratory Animal Research).

### Materials

All experimental events took place in operant conditioning chambers purchased from Med Associates, Inc. (St. Albans, VT, United States). Each chamber contained a houselight, a retractable response lever, a stimulus light located above the response lever, and a guillotine door leading to a smaller, adjacent compartment containing either a social or non-social stimulus. The two compartments were further separated by a metal screen that allowed rats in adjacent compartments full visual, auditory, and olfactory contact, as well as limited tactile contact with one another, but prevented each subject from traversing to the opposite compartment. Each chamber was housed within a larger, sound-attenuating cabinet, and white noise was continuously present during training and testing. Subjects self-administered cocaine intravenously through a Tygon tube surrounded by a stainless-steel spring connected to a swivel at the top of the cage and an infusion pump located outside of the cage.

Cocaine HCl was generously supplied by the National Institute on Drug Abuse (Research Triangle Institute, Research Triangle Park, NC, United States) and dissolved in sterile saline for intravenous administration.

### Lever-Press Training

One week after arrival, rats were restricted to 90% of their free-feeding body weight and trained to lever press using food reinforcement. These training sessions were conducted in operant conditioning chambers that were different from those that would later be used for drug self-administration, with a different configurational arrangement and located in a different testing room. During training, lever presses were reinforced with a single, 45-mg grain pellet on a fixed ratio (FR1) schedule of reinforcement. Sessions terminated once 40 reinforcers had been earned or 2 h elapsed, whichever occurred first. Training ended when a rat earned 40 reinforcers during any four training sessions, and all rats met this criterion within one week.

### Surgery

After lever-press training, rats were implanted with intravenous catheters into the right jugular vein under isoflurane anesthesia. Rats were treated with ketoprofen immediately after surgery and at 12-h intervals for 2 days. Wounds were treated with topical antibiotic immediately after surgery and daily for 2 days. Catheters were flushed daily with heparinized saline to maintain patency and ticarcillin to prevent infection.

### Social Partnering

One day before self-administration training commenced, all rats were paired with a stimulus rat of the same sex and age but had not undergone surgery. A single partnering session occurred in which the two rats were placed together in a clean, neutral cage and allowed unlimited social interaction for 15 min. One stimulus rat was typically partnered with at least 2 rats that would subsequently be trained for drug self-administration.

### Cocaine Self-Administration Training

Self-administration training began approximately one week after surgery and one day following social partnering. Training sessions were conducted in the operant conditioning chambers containing the guillotine door with adjacent side compartment; however, the guillotine door was not operational during these sessions, and no stimulus was placed in the adjacent compartment. Each session began with illumination of the house light, insertion of the lever inside the chamber, activation of the stimulus light above the response lever, and a non-contingent infusion of cocaine (0.5 mg/kg). For the remainder of the session, cocaine was available on an FR1 schedule of reinforcement. Each lever press produced an infusion of 0.5 mg/kg cocaine, retracted the response lever, and turned off the stimulus light above the lever. After 30 s, the lever was inserted back into the chamber, and the stimulus light above the lever was illuminated again. All sessions terminated automatically after 60 min. Training continued in this manner for five consecutive days.

### Introduction of the Social/Non-social Stimulus

After 5 days of training, contingencies were changed, and each lever press produced 0.5 mg/kg cocaine, retracted the response lever, turned off the stimulus light above the lever, and opened the guillotine door to the adjacent side compartment. Consequently, each cocaine infusion was simultaneously reinforced by 30-s exposure to either the social or non-social stimulus located in the adjacent compartment. After 30 s, the guillotine door closed, the stimulus light turned off, and the lever was reinserted into the chamber. Rats assigned to the social and non-social conditions were matched based on the number of reinforcers obtained during the 5 days of testing. Rats that were used for partnering (see above) were used as the social stimuli, such that each partner was assigned to a subject with which it had previous contact during the partnering session. A clean, black-and-white athletic sock of similar size and coloring as a Long-Evans rat served as the non-social stimulus. These conditions remained in effect for 10 consecutive days. Similar to training, all sessions were 60 min in duration.

### Dose-Effect Curve Determinations

After 10 sessions of exposure to the social/non-social stimuli, a cocaine dose-effect curve was determined in both the social and non-social groups. In these sessions, the dose of cocaine available in each infusion changed each day, such that a 100-fold dose range was examined (0.01–1.0 mg/kg). Five doses were tested in a pseudorandom order with the stipulation that no more than two ascending or descending doses could be tested in a row. In addition, a saline substitution test was conducted in which each infusion contained the vehicle.

Following an initial determination of the cocaine dose-effect curve, we tested whether reducing the magnitude of the social/non-social stimuli would reduce their reinforcing effects on cocaine intake. To this end, the duration in which the guillotine door was open was reduced from 30 to 5 s, thus decreasing the duration of access to the social and non-social stimuli. After 5-s exposure to the social/non-social stimulus, the guillotine door closed, but the response lever remained retracted for an additional 25 s to keep all other temporal parameters consistent across conditions. The cocaine-dose effect curve was then redetermined (and a saline substitution tested was reconducted) under the conditions described above over six consecutive sessions.

### Social/Non-social Stimulus Switch

Following redetermination of the cocaine dose-effect curve, the two groups functionally switched conditions, such that each rat originally assigned a social partner was now assigned a non-social stimulus (i.e., black-and-white athletic sock), and each rat originally assigned a non-social stimulus was assigned a social partner. Test sessions began on the following day with rats in their new group assignments, and cocaine self-administration was reinforced with 30-s access to the social or non-social stimulus. All other conditions were identical to those described above. The dose of cocaine (0.5 mg/kg/infusion) was held constant, and data were collected across 10 consecutive sessions.

### Data Analysis

A total of 21 rats (social: *n* = 12; non-social: *n* = 9) contributed to the acquisition and dose-response analyses. Three rats (original social: *n* = 2; original non-social = 1) lost catheter patency following the stimulus switch and were not included in the data analysis from the last 10 days of testing.

Data obtained during acquisition were analyzed *via* two-way, mixed-factor ANOVA, with group (social vs. non-social) serving as the between-subjects factor and session serving as the repeated-measure. Data from the dose-response analyses were analyzed *via* two-way, mixed-factor ANOVA, with group serving as the between-subjects factor and dose serving as the repeated-measure. Data from saline substitution tests were analyzed *via* independent-samples *t*-tests. Data obtained during the stimulus switch were analyzed *via* two-way, mixed-factor ANOVA, with group serving as the between-subjects factor and session serving as the repeated-measure. *Post hoc* tests were conducted *via* independent-samples or paired-samples *t*-tests where appropriate, following by the Holm’s-Bonferroni correction for multiple comparisons. All statistical tests were two-tailed, and the alpha value was set to 0.05.

## Results

**FIGURE 1:**
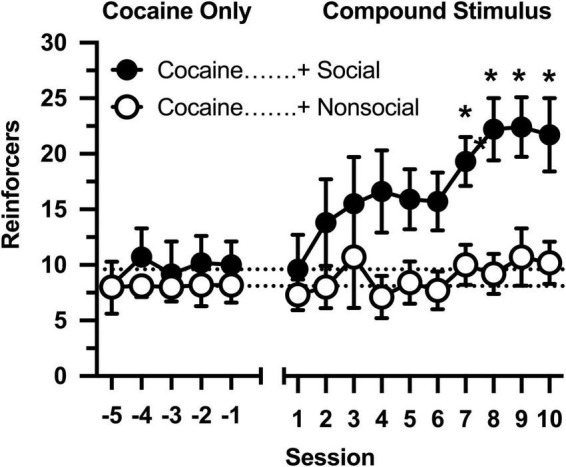
Number of reinforcers obtained during daily, 60-min test sessions. Data reflect the mean (SEM) number of cocaine infusions from rats assigned to the social (“+ Social,” filled symbols; *n* = 12) and non-social (“+ Non-social,” open symbols; *n* = 9) groups. For the first five sessions (Sessions –5 to –1), responding was maintained only by cocaine (0.5 mg/kg). For the following 10 sessions (Sessions 1 to 10), responding was maintained by cocaine and 30-s access to either a social (sex- and age-matched partner) or non-social (black-and-white athletic sock) stimulus. Dashed lines originating at the X-intercept reflect the average number of cocaine infusions during the first five sessions (social: 9.6 infusions; non-social: 8.1 infusions). Asterisks indicated significant differences between social and non-social groups.

All rats responded on the first day of cocaine availability, receiving approximately 9 infusions per session (Figure 1: Sessions –5 to –1). The number of reinforcers was consistent over 5 consecutive days in which responding was maintained by cocaine, and no differences were observed between rats that would later by assigned to the social and non-social conditions (no main effect of group, main effect of session, or group × session interaction was observed).

Cocaine intake was selectively impacted by the social stimulus when operant contingencies changed and responding resulted in opening the guillotine door (Figure 1: Sessions 1 to 10). Cocaine intake progressively increased in rats in the social partner condition [main effect of session: *F*(9,171) = 4.691, *p* < 0.001], resulting in significantly greater cocaine intake in this group relative to rats in the non-social condition [main effect of group: *F*(1,19) = 5.530, *p* = 0.030]. In contrast, cocaine intake did not increase in the non-social condition [group × session interaction: *F*(9,171) = 1.984, *p* = 0.044], resulting in significant between-group differences in cocaine intake during sessions 6 through 10.

**FIGURE 2:**
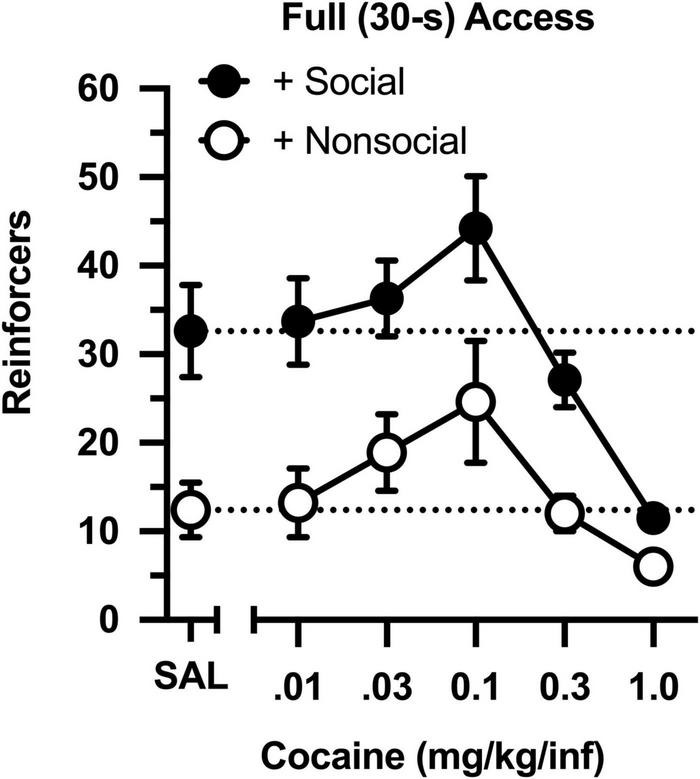
Number of reinforcers obtained during daily, 60-min test sessions under full (30-s) access conditions. Data reflect the mean (SEM) number of infusions from rats assigned to the social (“+ Social,” filled symbols; *n* = 12) and non-social (“+ Non-social,” open symbols; *n* = 9) groups. Responding was maintained by various doses of cocaine (0.01–1.0 mg/kg/inf) and either a social (sex- and age-matched partner) or non-social (black-and-white athletic sock) stimulus. Dashed lines originating at the X-intercept reflect the average number of infusions during a saline substitution test (SAL).

Across a 100-fold dose range, cocaine intake was characterized by an inverted, U-shaped dose-effect curve in both groups (Figure 2), with ascending and descending limbs converging at 0.1 mg/kg [main effect of dose: *F*(4,76) = 15.851, *p* < 0.001]. Across all doses, cocaine intake ranged from 2- to 3-fold higher in the social group than in the non-social group [main effect of group: *F*(1,19) = 13.421, *p* = 0.002]. Importantly, responding was greater in the social than non-social group during a saline substitution test [*t*(19) = 9.661, *p* = 006], reflecting the differential reinforcing effects of the social/non-social stimuli in the absence of cocaine.

**FIGURE 3:**
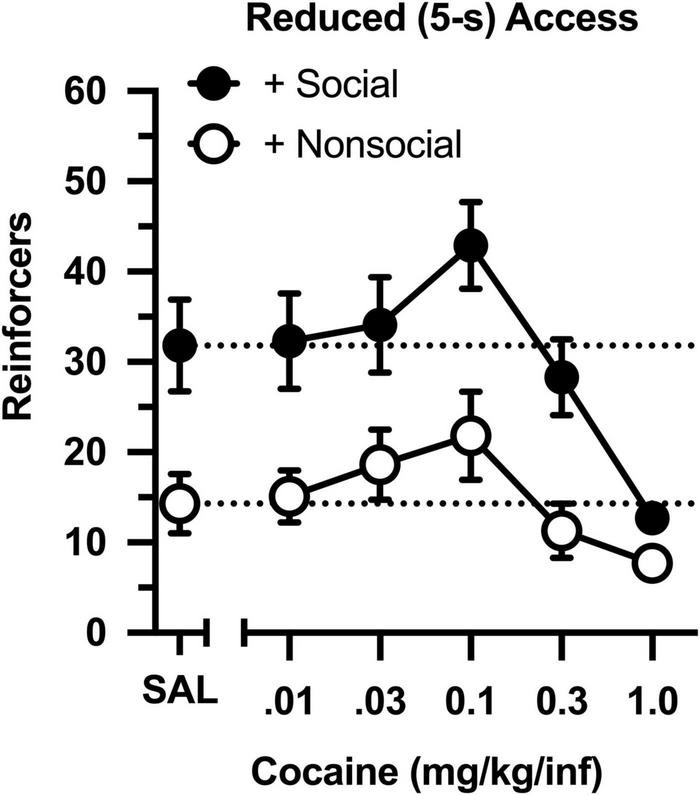
Number of reinforcers obtained during daily, 60-min test sessions under reduced (5-s) access conditions. Data reflect the mean (SEM) number of infusions from rats assigned to the social (“+ Social,” filled symbols; *n* = 12) and non-social (“+ Non-social,” open symbols; *n* = 9) groups. Responding was maintained by various doses of cocaine (0.01–1.0 mg/kg/inf) and either a social (sex- and age-matched partner) or non-social (black-and-white athletic sock) stimulus. Dashed lines originating at the X-intercept reflect the average number of infusions during a saline substitution test (SAL).

The dose-effect curve was redetermined after reducing the magnitude of the social and non-social stimuli (Figure 3). Reducing the duration of door opening from 30 to 5 s did not impact the dose-effect curve in either group (compare Figures 2 and 3). Similar to the original determination, cocaine intake was characterized by an inverted, U-shaped dose-effect curve [main effect of dose: *F*(4,76) = 13.793, *p* < 0.001], and intake ranged from 2- to 3-fold greater in the social than non-social group [main effect of group: *F*(1,19) = 11.647, *p* = 0.003]. Responding was also greater in the social than non-social group during a saline substitution test [*t*(19) = 5.191, *p* = 034].

**FIGURE 4:**
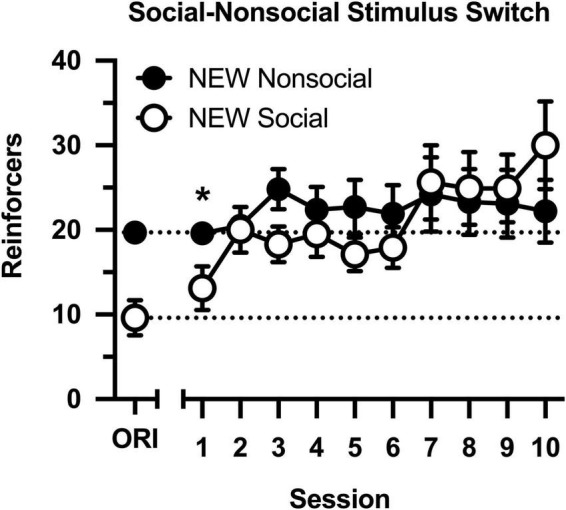
Number of reinforcers obtained during daily, 60-min test sessions. Data reflect the mean (SEM) number of cocaine infusions from rats newly switched to the non-social (filled symbols; *n* = 10) and social (open symbols; *n* = 8) groups. During all sessions, responding was maintained by cocaine (0.5 mg/kg) and 30-s access to either a social (sex- and age-matched partner) or non-social (black-and-white athletic sock) stimulus. Dashed lines originating at the X-intercept reflect the average number of cocaine infusions during Session 10 of the groups’ original assignments (ORI). Asterisk indicates significant difference between social and non-social groups.

Following determinations of the cocaine dose-effect curve, conditions were switched (Figure 4), such that rats in the original social condition were switched to the non-social condition (i.e., partners were switched for socks), and rats in the original non-social condition were switched to the social condition (i.e., socks were switched for partners). Cocaine intake increased across 10 consecutive sessions [main effect of session: *F*(9,144) = 4.326, *p* < 0.001], and this was driven by selective increases in intake in the new social group [group × session interaction: *F*(9,144) = 2.645, *p* = 0.007]. Cocaine intake was significantly less in the new social group during the first session, but the groups did not differ during the final nine sessions. Notably, cocaine intake remained high and stable in the new non-social group, reflecting the persisting effects of exposure to the social stimulus.

## Discussion

This study used a novel preclinical model to determine whether access to a social partner positively reinforces cocaine self-administration and thus facilitates cocaine intake. We found that contingent access to a social partner, but not a non-social stimulus, rapidly increases cocaine intake resulting in a 2- to 3-fold escalation of cocaine intake relative to both baseline and control conditions. These data emphasize the importance of social peers in the escalation of drug intake and demonstrate the role of social reinforcement in vulnerability to drug abuse.

Responding was under control of both reinforcers. Lever pressing was readily established with cocaine, and cocaine intake in both groups was characterized by an inverted, U-shaped dose-effect curve, which is typical of responding maintained by cocaine on simple FR schedules of reinforcement (Lynch and Carroll, 2001). Lever pressing increased rapidly when the social stimulus was introduced, both at the beginning of the study in the original social group, and toward the end of the study in the non-social group after the stimulus switch. Moreover, twice as many reinforcers were obtained during a saline substitution test in the social group than the non-social group, revealing the effects of the social contingency in the absence of cocaine. Finally, it is notable that high doses of cocaine markedly reduced responding relative to vehicle (i.e., saline) control values, thus functioning as a positive punisher to reduce responding otherwise maintained by social contact.

Recent studies have used several experimental designs to examine how social contact interacts with drug reward. For instance, studies using the conditioned place preference procedure report that social contact increases the conditioned rewarding effects of a drug if the stimuli are conditioned in the same compartment (Thiel et al., 2008; Reyna et al., 2021). In contrast, social contact can block the conditioned rewarding effects of a drug if social contact is provided exclusively in the opposite compartment (Fritz et al., 2011; Zernig and Pinheiro, 2015). Drug self-administration studies demonstrate the presence of a partner that is also self-administering drugs enhances drug intake, whereas the presence of a partner that is not self-administering drugs inhibits drug intake (Smith, 2012; Robinson et al., 2016). Unlike the present study, the presence of the partner was not contingent on drug intake in those previous studies. Finally, a social partner can inhibit drug intake in a discrete-choice procedure when selection of that partner specifically excludes drug delivery (Venniro et al., 2018, 2019).

Several behavioral mechanisms have been used to explain how drug use may be established and maintained among peers, and they include classic learning phenomena such as imitation/modeling, social reinforcement, emulation, social facilitation, local enhancement, stimulus enhancement, and reinforcement enhancement (Strickland and Smith, 2014). Although this study was not designed to systematically test all possible mechanisms, the current data may be used in conjunction with data collected previously to rule out several possibilities. Explanations based on imitation/modeling, emulation, local enhancement, and stimulus enhancement require a model to be engaged in behavior that increases a subject’s attention to (or engagement with) a discriminative stimulus, behavioral action, or reinforcing stimulus. Social partners did not have access to either response levers or intravenous cocaine, thus limiting their ability to increase the salience of any component of the lever-press/cocaine infusion contingency. Social facilitation or reinforcement enhancement represent two potential explanations for these findings, but multiple studies report the presence of a non-intoxicated social partner reliably *decreases* drug intake across a range of experimental conditions (Smith, 2012; Peitz et al., 2013; Smith et al., 2014; Robinson et al., 2016, 2017; Venniro et al., 2018, 2019, 2021). The experimental design of the present study specified that social contact was contingent on pressing a response lever and self-administering cocaine; consequently, social reinforcement reflects the most parsimonious explanation for the observed findings, especially after other explanations are ruled out. Importantly, the non-social (i.e., negative control) condition ruled out the possibility that extraneous components of the reinforcing stimulus (e.g., guillotine door opening, presence of novel stimulus) was responsible for the escalation of cocaine intake.

One observation that questions the role of social reinforcement in the maintenance of elevated responding is that cocaine intake was not responsive to either a reduction in the magnitude of the social stimulus (i.e., a decrease in the duration of social access) or the removal of the social stimulus (i.e., replacing a social partner for an athletic sock). Previous studies examining social reinforcement reveal that greater social contact (visual plus partial physical contact > only visual contact; Angermeier, 1960) and greater social deprivation (longer deprivation > shorter deprivation; Achterberg et al., 2016) increase the reinforcing effects of social contact. We do not know of any studies that manipulated the duration of social contact when measuring social reinforcement; however, studies using conditioned place preference report that longer durations of social contact do not lead to greater reward (10 min contact = 30 min contact; Thiel et al., 2008). In the present study, the elevated levels of cocaine intake in the social group were remarkably stable and not responsive to further manipulations, including the removal of the social stimulus. These data suggest that other behavioral mechanisms were also recruited to produce long lasting increases in drug intake that were resilient to further behavioral manipulations. This observation is of translational concern because it indicates that once drug use increases in response to social reinforcement from others, it will persist even in the absence of those individuals and may be less responsive to some first-line psychosocial interventions.

Collecting additional measures of behavior would help identify the mechanisms contributing to the differences in responding between social and non-social conditions. For instance, responding on an inactive lever would reveal the degree to which a social partner increased general levels of activity. Alternatively, the presence of a second active lever that resulted in only cocaine delivery (or only social access) would determine the preference for each stimulus individually relative to the compound stimulus. Furthermore, the presence of a second active lever that resulted in a different stimulus (e.g., sucrose, electric shock) would determine the extent to which social contact could differentially reinforce behaviors maintained at high rates (e.g., food-maintained responding) versus low rates (e.g., shock-maintained responding). Our hypothesis would be that the effects of social reinforcement would generalize broadly to other behaviors, particularly those maintained at low rates (Herrnstein, 1961).

We did not collect within-session patterns of responding or videotape the rats during test sessions. In the former case, we made a programming error that prevented the creation of cumulative records; in the latter case, video cameras were not available to us at the time of data collection. Consequently, we do not know the extent to which the subjects interacted with the social and non-social stimuli, nor do we know the nature of the interaction (e.g., active exploratory, passive avoidance). Relating the temporal pattern of lever-pressing to behaviors emitted during the 30-s stimulus presentation would provide further insight into the mechanisms responsible for the stimulus control of cocaine self-administration.

Females should be tested in future iterations of this model, especially when considering that male and female adolescents respond differently to social pressure on measures of drug use (Dick et al., 2007; Schulte et al., 2009; Mason et al., 2014; McMillan et al., 2018). An additional area ripe for investigation involves the effects of male-female interactions on measures of drug intake. There is nothing about the present model that would prevent the use of sexually mature male and female subjects as social partners, including female subjects during various phases of behavioral estrous. Although sex is frequently included as a biological variable in behavioral assays, interactions with the opposite sex are frequently ignored outside of studies specifically examining courtship/mating behavior.

The experimental design intentionally limited cocaine intake by restricting cocaine availability. Levers were retracted during periods of social/non-social stimulus presentation to encourage engagement with the reinforcing stimuli, and sessions were limited to 60 min to prevent satiation of the social stimulus. Limited access procedures do not model problematic patterns of drug intake that characterize addictive behavior (Smith, 2020), so data from this study are more translationally relevant to social drug use than drug abuse and addiction. This study also only examined the effects of social reinforcement on intravenous cocaine self-administration. Future studies must examine other drugs and routes of administration, particularly drugs and routes of administration that are more relevant to adolescent and young adult populations (e.g., oral alcohol consumption, inhaled nicotine/cannabinoid consumption).

The most obvious translational implication of this study is that social reinforcement escalates cocaine use, identifying it as a vulnerability factor for drug abuse and addiction. Prevention programs for at-risk groups (e.g., younger adolescents involved in predelinquent behavior) should target group norms that are permissive or even encouraging of drug use. Alternatively, it is equally likely that abstinence-related behaviors (e.g., attending school, involvement in religious and community activities, showing up for work) could be differentially reinforced by non-drug rewards, including social activities with abstinent individuals. Community reinforcement programs often make use of social reinforcement (e.g., integrating the person in a social network that engages in non-drug recreational activities) to encourage abstinence, and these programs have higher success rates than standard-care control groups (see review by Meyers et al., 2011). This could easily be modeled in future studies by using a differential reinforcement of other behavior (DRO) schedule in which social reinforcement is contingent on behavior that excludes drug self-administration. Indeed, previous studies have successfully modeled “voluntary abstinence” by providing rats with a discrete choice between drug delivery and social contact after a history of drug self-administration. Those studies reveal that social contact decrease drug intake, prevents the incubation of craving, and decreases measures of relapse in both males and females (Venniro et al., 2017, 2019).

## Conclusion

This study describes a preclinical model in which cocaine self-administration is positively reinforced by contingent access to either a social (age- and sex-matched rat) or non-social (black-and-white athletic sock) stimulus. Contingent access to a social partner rapidly increased cocaine intake 2- to 3-fold across an extensive dose range. These increases in cocaine intake were persistent over time and resistant to later reductions in and removal of the social stimulus. Data collected in this model suggest that social reinforcement may contribute to the escalation of drug intake among peers and represents a vulnerability factor that may be particularly resistance to psychosocial interventions once established.

## Data Availability Statement

The raw data supporting the conclusions of this article will be made available by the authors, without undue reservation.

## Ethics Statement

The animal study was reviewed and approved by Davidson College Animal Care and Use Committee.

## Author Contributions

MS conceived the study and wrote the manuscript. HC, AG, and JS collected the data. MS and JS analyzed the data. All authors approved the final draft and are accountable for the work.

## Conflict of Interest

The authors declare that the research was conducted in the absence of any commercial or financial relationships that could be construed as a potential conflict of interest.

## Publisher’s Note

All claims expressed in this article are solely those of the authors and do not necessarily represent those of their affiliated organizations, or those of the publisher, the editors and the reviewers. Any product that may be evaluated in this article, or claim that may be made by its manufacturer, is not guaranteed or endorsed by the publisher.
